# Tuning the Direct and Indirect Excitonic Transitions of *h*-BN by Hydrostatic Pressure

**DOI:** 10.1021/acs.jpcc.1c02082

**Published:** 2021-06-03

**Authors:** Alfredo Segura, Ramon Cuscó, Claudio Attaccalite, Takashi Taniguchi, Kenji Watanabe, Luis Artús

**Affiliations:** † Departamento de Física Aplicada-ICMUV, Malta-Consolider Team, Universitat de València, 46100 Burjassot, Spain; ‡ GEO3BCN-CSIC, Consejo Superior de Investigaciones Científicas, C. Lluís Solé i Sabarís s.n., 08028 Barcelona, Spain; § Aix Marseille Université, CNRS, CINaM UMR 7325, Campus de Luminy, Case 913, 13288 Marseille, France; ∥ International Center for Materials Nanoarchitectonics, National Institute for Materials Science, 1-1 Namiki, Tsukuba 305-0044, Japan; ⊥ Research Center for Functional Materials, National Institute for Materials Science, 1-1 Namiki, Tsukuba 305-0044, Japan

## Abstract

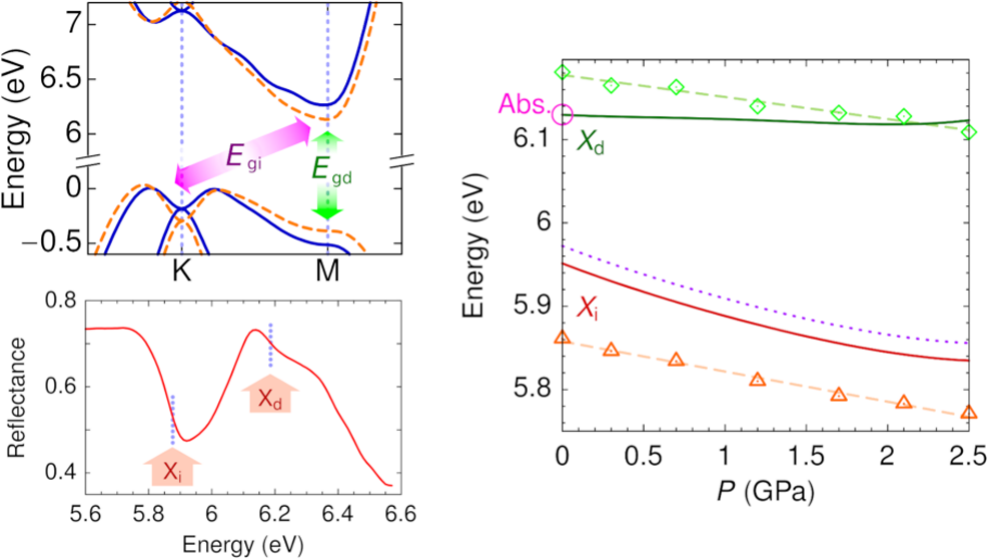

The pressure dependence of the direct and indirect bandgap transitions of hexagonal boron nitride is investigated using optical reflectance under hydrostatic pressure in an anvil cell with sapphire windows up to 2.5 GPa. Features in the reflectance spectra associated with the absorption at the direct and indirect bandgap transitions are found to downshift with increasing pressure, with pressure coefficients of −26 ± 2 and −36 ± 2 meV GPa^–1^, respectively. The *GW* calculations yield a faster decrease of the direct bandgap with pressure compared to the indirect bandgap. Including the strong excitonic effects through the Bethe–Salpeter equation, the direct excitonic transition is found to have a much lower pressure coefficient than the indirect excitonic transition. This suggests a strong variation of the binding energy of the direct exciton with pressure. The experiments corroborate the theoretical predictions and indicate an enhancement of the indirect nature of the bulk hexagonal boron nitride crystal under hydrostatic pressure.

## Introduction

Hexagonal boron nitride (*h*-BN) is emerging as an exceptional material with a multitude of applications in nanophotonics, quantum photonics, and deep-UV optoelectronics.^1−3^ Its unique properties include an ultrawide bandgap (∼6 eV), very high thermal conductivity and stability, a lamellar honeycomb structure similar to graphene, a natural optical hyperbolic behavior, and an unusually bright deep-UV emission. This bright emission was initially attributed to a direct transition,^4^ despite the observation of a Stokes shift between absorption and emission and a fine structure in the emission spectra. A long-standing controversy has existed over the nature of exciton transitions in *h*-BN,^5,6^ which was finally resolved by precise measurements on high-purity samples revealing that the fine structure in the emission spectra could be explained by phonon-assisted transitions from the conduction band minimum at the *M* point to the valence band maximum around the *K* point.^7^ This finding has stimulated theoretical work on the fundamental properties of excitons in *h*-BN. The indirect nature of the strongly bound lowest-energy exciton and the presence of a direct exciton at a slightly higher energy have been calculated by *ab initio* methods,^8^ and the lowest-energy exciton was then measured in electron-loss spectroscopy experiments.^9^ The large intensity of the phonon-assisted transitions in *h*-BN has been accounted for by Green’s function calculations with the inclusion of electron–phonon coupling by means of a finite-difference approach.^10,11^

Important physical properties of two-dimensional (2D) layered materials such as van der Waals interactions, crystal structure, and electronic band structure can be tailored by strain. This has prompted a burgeoning interest in high-pressure studies of 2D materials and a plethora of works that have demonstrated the potential of pressure for controlling a wide range of physical properties including lattice distortions and phase transitions, phonon dynamics, metallic or superconducting states, charge transfer and doping, and optical emission.^12^ The modulation of the bandgap using hydrostatic pressure is a powerful probe into the strong coupling between mechanical and optical properties of layered materials.^13^ A remarkable bandgap opening of 2.5 eV has been recently achieved in a semiconducting state of compressed trilayer graphene by tuning the interlayer hybridization.^14^

The vibrational and structural properties of *h*-BN under pressure have been recently investigated.^15−18^ However, despite its emerging importance, experimental studies of the hydrostatic pressure behavior of the *h*-BN electronic band structure are scarce.^19^ In contrast, strain engineering of the band structure has been thoroughly explored in transition-metal dichalcogenides.^20,21^ The strong enhancement of the photoluminescence intensity observed in few-layer WSe_2_ under uniaxial strain was attributed to a strain-induced indirect-to-direct bandgap transition.^20^ Later studies under hydrostatic pressure revealed a pressure-induced *K–*Λ crossing in monolayer WSe_2_ and therefore a transition from direct to indirect character of the optical emission.^22^ Both indirect (Λ–*K*) and direct (*K*–*K*) bandgap transitions were observed in bilayer WSe_2_. The pressure coefficient of the direct interband transition is positive and of similar magnitude for monolayer and bilayer WSe_2_. In contrast, the pressure coefficient of the indirect transition is negative and it is significantly larger for bilayer WSe_2_, thus enhancing the indirect character of the optical emission in bilayer WSe_2_ with increasing pressure.

With the aim of investigating the modulation of direct and indirect transitions by hydrostatic pressure in *h*-BN and the possibility of influencing its indirect character, in this article, we report optical reflectance and absorption studies on *h*-BN for hydrostatic pressures up to 2.5 GPa. These are the first experimental detailed studies of band structure shift under pressure in this layered material. They provide insight into the different nature of the intralayer and interlayer interactions in *h*-BN and serve as a benchmark for theoretical calculations. As predicted by *ab initio* calculations, the bandgap is observed to narrow with increasing pressure, and the pressure coefficients for the direct and indirect transitions are determined.

## Experimental and Modeling Methods

The high-quality single crystals were synthesized at 4.5 GPa and 1500 °C using barium boron nitride as a solvent in a modified belt-type high-pressure and high-temperature apparatus in a dry nitrogen atmosphere. The samples and the solvent were encapsulated in a molybdenum sample chamber inside a nitrogen-purged glove box. After the high-pressure–high-temperature cycle, the molybdenum sample chamber was dissolved using hot aqua regia to obtain the *h*-BN crystals. The details of the growth process are described elsewere.^23^

A sample of thickness 6.7 μm was cleaved from *h*-BN single-crystalline platelets to perform the reflectance measurements under pressure. A very thin sample of thickness around 50 nm was also obtained to record the absorption spectra. Measurements at high pressure were carried out in a gasketed membrane anvil cell using methanol–ethanol–water (16:3:1) as a pressure transmitting medium. To gain access to the spectral region of *h*-BN bandgap, sapphire anvils were used in the high-pressure cell. A noncommercial, all-reflecting microscope optical bench was employed for the optical measurements, with a deuterium lamp as the excitation source.

The experimental results were analyzed on the basis of density functional theory (DFT) calculations with projector augmented wave (PAW) pseudopotentials^24^ and the wdW-DF3 van der Waals functional.^25^ Unit cell relaxation was carried out at every pressure, using a cutoff of 100 Ry on the wavefunction and a shifted 18 × 18 × 6 *k*-point grid. The quasi-particle band structure was calculated within the *GW* framework, with self-consistency on both *G* and *W* eigenvalues.^26^ The strong excitonic effects in the optical response were taken into account using the Bethe–Salpeter equation.^27^ The calculation parameters are the same as those used in ref (3). A rigid shift of 196 meV independent of pressure has been applied to the calculated conduction band structure to bring the calculated absorption peak in full accord with the absorption peak determined by synchrotron radiation experiments.^3^

## Results and Discussion

**Figure 1:**
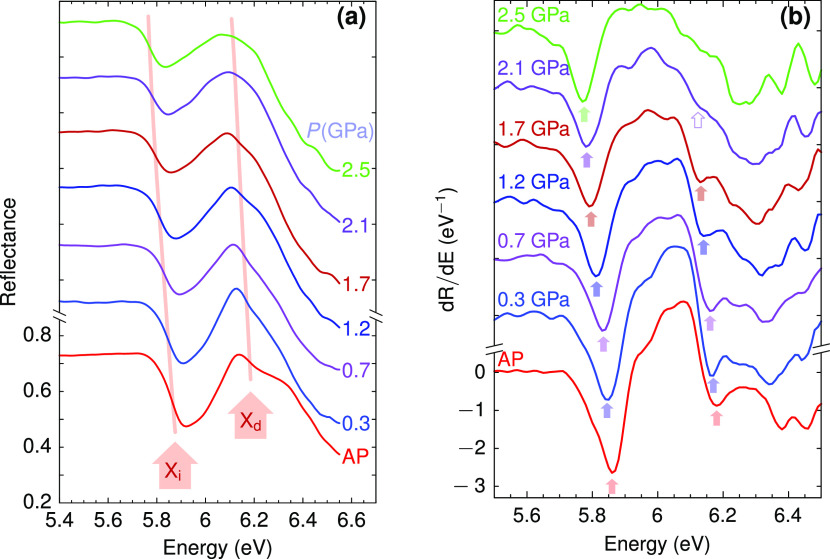
(a) Reflectance spectra of *h*-BN for pressures up to 2.5 GPa. The traces have been vertically shifted by 0.2 steps for clarity. The vertical lines indicate the shift with pressure of the features associated with the direct (*X*_d_) and indirect (*X*_i_) excitonic transitions. (b) Derivative of the reflectance spectra. The traces have been vertically shifted for clarity. The arrows indicate the minima of d*R*/d*E* corresponding to the excitonic transition.

Figure 1a shows the reflectance spectra measured for pressures up to 2.5 GPa. The reflectance spectra typically display a sudden drop in the 5.7–5.9 eV range and a subsequent broad structure in the 6.0–6.4 eV range. Both features show a redshift with increasing pressure, as indicated by the vertical lines in Figure 1a. The reflectance drop is a consequence of the absorption onset at the indirect excitonic transition, which strongly suppresses the contribution of the reflection at the bottom surface of the *h*-BN flake. A similar self-absorption effect is expected to occur at the direct exciton transition. To accurately determine the excitonic transition energies, we have considered the derivative of the reflectance spectra. Figure 1b displays the derivative of the reflectance spectra at different pressures. The traces clearly show two minima, indicated by fat arrows, which correspond to the *X*_i_ and *X*_d_ features of the reflectance spectra identified in Figure 1a. While the derivative traces exhibit a deep minimum at the *X*_i_ transition for all pressures, the minimum associated with the *X*_d_ transition is less pronounced and tends to fade away at higher pressures as a consequence of the broadening of the reflectance band [see Figure 1a]. At 2.1 GPa, an inflection in d*R*/d*E* can still be seen at the expected *X*_d_ energy, indicated by an empty arrow in Figure 1b. The location of this feature is determined with the aid of the second derivative of the reflectance. At the highest pressure studied (2.5 GPa), the broad reflectance band is essentially featureless, and the *X*_d_ energy is roughly estimated as the center of the broad band.

**Figure 2:**
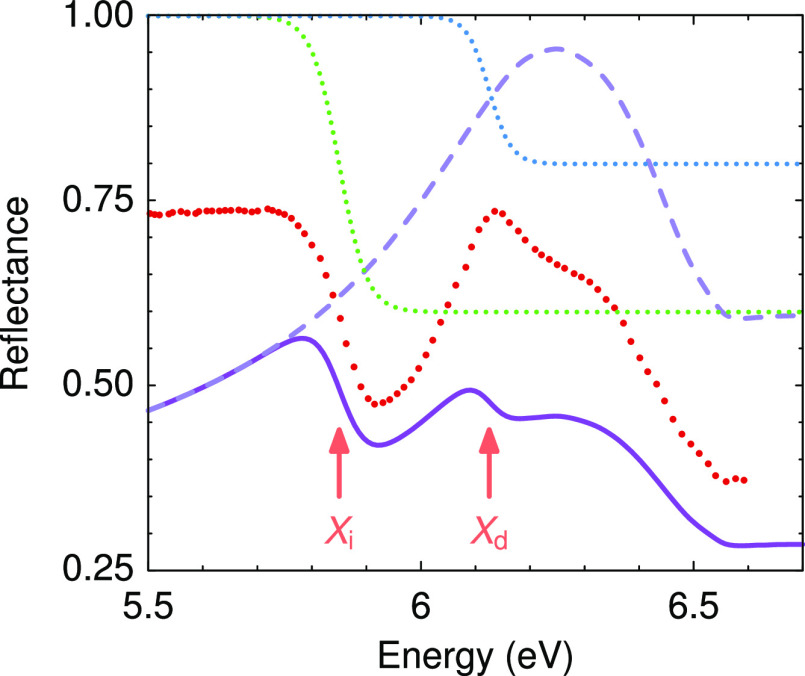
Calculated reflectivity spectrum associated with the direct excitonic transitions (dashed line), compared with the experimental reflectance measurement (red dots). The blue and green sigmoidal dotted lines account for, respectively, the drop of reflectance due to the absorption at the direct and indirect excitonic energies suppressing the reflection at the bottom interface. The solid line is the resulting calculated reflectance after taking into account the self-absorption effect.

In Figure 2, we compare the experimental reflectance spectra at ambient pressure (red dots) with the reflectivity obtained from our *ab initio* calculations for semi-infinite bulk *h*-BN (dashed line). The calculated trace displays a single broad maximum centered at ∼6.25 eV, above the direct excitonic transition. This calculated trace differs from the measured reflectance spectrum in the thin *h*-BN platelet because of the signal reflected at the back interface, which is modulated by the absorption in the sample. To account for the effect of self-absorption, we have considered a sigmoidal-like drop of the reflected intensity centered at the calculated excitonic transitions, as depicted by the dotted lines. By applying these additional reflectance drops to the calculated reflectivity spectrum, we obtain the trace plotted as a solid line, which clearly shows characteristic features associated with the indirect and direct excitonic transitions and resembles the experimental reflectance spectrum. At energies below the indirect excitonic transition, the increase of the top surface reflection due to the increase of the material reflectivity is compensated by the decrease of the back interface contribution resulting from residual absorption in the sample. This analysis supports the use of the derivative of the reflectance [shown in Figure 1b] to determine accurate values for the excitonic transition energies.

**Figure 3:**
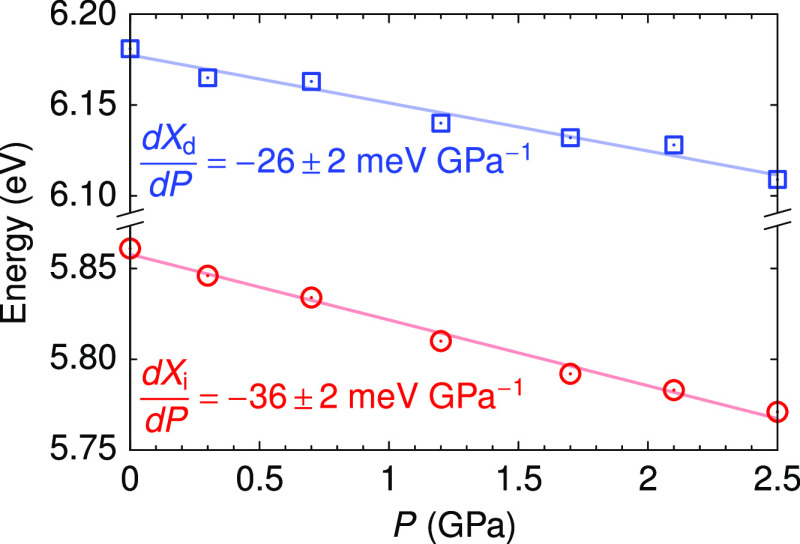
Pressure dependence of the *X*_d_ and *X*_i_ energies as determined from the reflectance spectra.

The direct and indirect excitonic transition energies thus obtained are plotted in Figure 3 for hydrostatic pressures up to 2.5 GPa. Both the direct and indirect excitonic transitions derived from the reflectance analysis exhibit a linear redshift with pressure, with pressure coefficients of −26 ± 2 and −36 ± 2 meV GPa^–1^, respectively. The pressure coefficient we find here for the indirect excitonic transition coincides with the pressure coefficient of the absorption edge given by Akamaru et al.,^19^ although the absorption edge energy reported in ref (19) was slightly higher and it was attributed to a direct gap.

**Figure 4:**
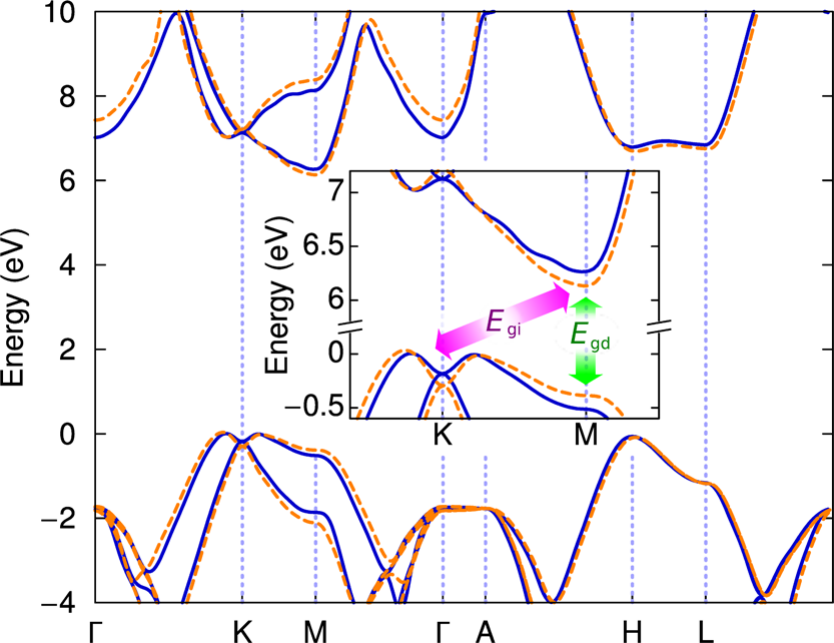
Electronic band structure calculated at the *GW* level. Solid lines are ambient pressure results, and dashed lines correspond to a hydrostatic pressure of 2.5 GPa. Inset: blow-up view of the band structure in the region of the indirect and direct bandgaps.

The electronic band structures at ambient pressure and at 2.5 GPa are plotted in Figure 4. The direct bandgap is located at the *M* point, whereas the indirect gap involves the valence band maximum close to the *K* point along the Γ–*K* line. As can be clearly seen in the inset, at higher pressures, the bandgap narrows along the *K*–*M* line. This is due to the increase of the interlayer interaction, which enlarges the band splitting along the *K*–*M* line. Contrarily, along the *H*–*L* line, the bands are essentially unaffected by the interaction with neighboring layers and they retain the monolayer structure.^28^ Notice that the intralayer interaction also changes with pressure, but this has little effect on the bandgap due to the extremely small variation of the *in-plane* lattice parameter *a*, which can be considered as constant in the pressure range studied.

**Figure 5:**
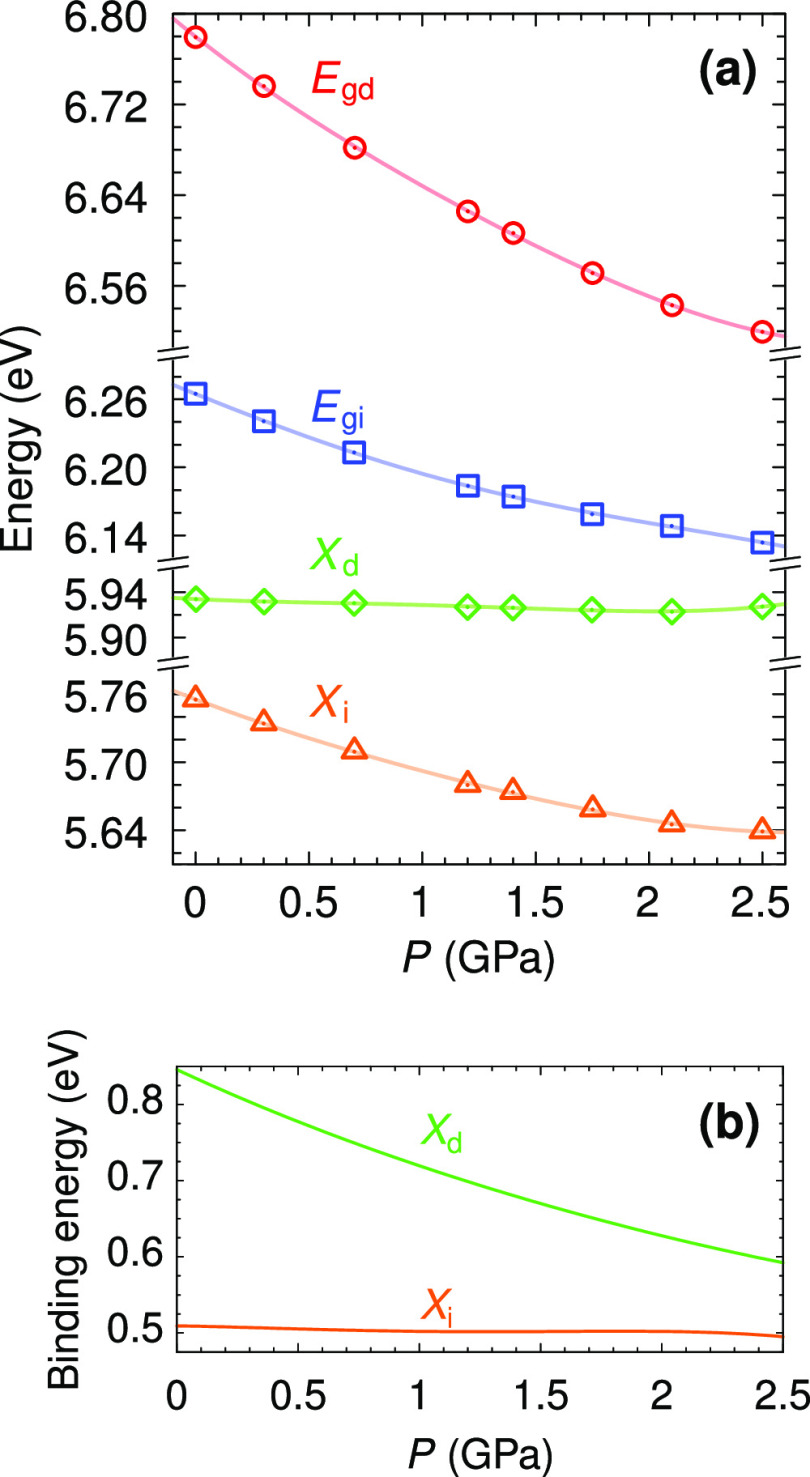
(a) Pressure dependence of the direct and indirect bandgaps and excitonic transitions of *h*-BN. The direct (*E*_gd_) and indirect (*E*_gi_) bandgaps were calculated at the *GW* level. The direct (*X*_d_) and indirect (*X*_i_) excitonic transitions were calculated at the *ab initio* many-body perturbation theory level (*GW* and Bethe–Salpeter equation). (b) Exciton binding energies as a function of pressure.

The results of the theoretical calculations at different hydrostatic pressures are reported in Figure 5a. The direct bandgap (*E*_gd_) shows a pronounced superlinear decrease with increasing pressure, as a consequence of the important reduction in the *c* lattice parameter and the consequent enhancement of the interlayer interaction. The increase of the interlayer interaction gives rise to an enlargement of the π band splitting around the *M* point, which results in a significant narrowing of the direct bandgap (see the inset in Figure 4). The indirect bandgap (*E*_gi_) also decreases with pressure to a lesser extent due to the lower pressure sensitivity of the valence band maximum close to *K*. When the electron–hole interaction is included, the resulting energy difference between direct (*X*_d_) and indirect (*X*_i_) exciton transitions becomes much smaller than the energy difference between *E*_gd_ and *E*_gi_. The electron–hole interaction enhances the electron localization close to the hole, which notably reduces the exciton dispersion.^8,30^ As a consequence of the flattening of the electron–hole dispersion by excitonic effects, the binding energies of direct and indirect excitons are different.^8^

As opposed to the large reduction in *E*_gd_ with pressure, the direct exciton transition is essentially unaffected by hydrostatic pressure. This is due to the fact that the direct exciton is formed by transitions around the *K* and *H* points of the Brillouin zone,^3^ which are only slightly affected by the external pressure.^28^ This different sensitivity to pressure implies that the exciton binding energy decreases significantly with pressure, as shown in Figure 5b. In contrast, the indirect excitonic transition shows a pressure dependence parallel to that of *E*_gi_ and hence, in this case, the binding energy remains constant up to the highest pressure investigated (2.5 GPa). The large calculated binding energies of 0.85 and 0.51 eV that we find, respectively, for the direct and indirect excitons at ambient pressure are consistent with previously reported values.^8,31,32^

**Figure 6:**
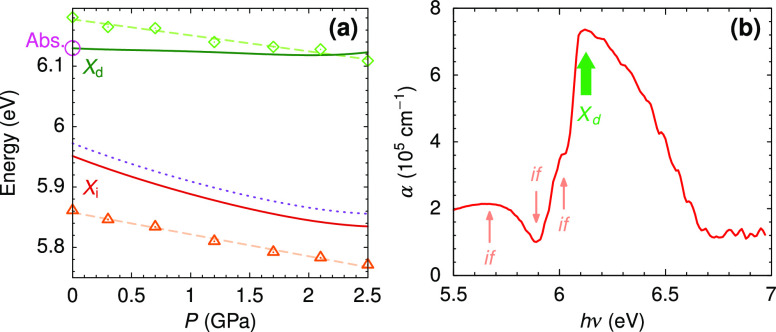
(a) Direct (*X*_d_, diamonds) and indirect (*X*_i_, triangles) excitonic transitions determined from the reflectance spectra compared with the *ab initio* theoretical calculations (solid lines). A rigid shift of 196 meV independent of pressure was applied to the theoretical results to bring them into accordance with the measured peak of the absorption spectrum (magenta circle). The dotted line represents the indirect transition absorption peak taking into account the energy of the ZA/ZO_1_ (*T*) assisting phonons.^29^ (b) Absorption spectrum at ambient pressure of a very thin *h*-BN sample (∼50 nm) in the spectral region of the direct excitonic transition. The arrows labeled *if* indicate structures corresponding to the Fabry–Perot interference fringe pattern of the 50 nm thick sample. The fat arrow indicates the direct excitonic transition.

In Figure 6a, we compare the experimental transition energies obtained from the reflectance measurements with the theoretical calculations. The energy of the direct transition determined from the reflectance data is corroborated by optical absorption measurements. Figure 6b shows the absorption spectrum at ambient pressure of a 50 nm thick *h*-BN sample in the spectral region of the direct excitonic transition. The absorption peak is observed around ∼6.12 eV, in reasonable agreement with the values observed in Figure 1a,b and coincident with previous ellipsometric measurements,^3^ thus validating the approach used to extract excitonic transition energies from the reflectance data. Since the calculated electronic transitions are affected by the underestimation of the quasi-particle bandgap and the neglect of vibrational renormalization,^33^ a small correction of ∼196 meV independent of pressure has been applied to the theoretical results to match the calculations with the experimental absorption peak at ambient pressure [magenta circle in Figure 6a]. It is worth noting that the calculations predict a fairly constant direct exciton transition energy, whereas the experimental points show a small decrease with pressure. This is not a contradictory observation, since the shape and width of the reflectivity peaks depend on both the energy position and on the intensity of the exciton.^34^ In the optical response calculations, it is found that the exciton dipole decreases with pressure. This also explains the redshift of the direct-exciton feature observed in the reflectance spectra. A broadening of the exciton absorption with increasing pressure, as suggested by the reflectance spectra of Figure 1a, could also lead to a redshift of the direct-exciton-related reflectance. On the other hand, the theoretical calculations tend to slightly overestimate the indirect transition energy by less than 2%. Nevertheless, the rate of decrease with pressure of the indirect excitonic transition energy, which is mainly governed by the lowering of the conduction band minima at *M* (see Figure 4), is consistent with the experimental results.

Both the theoretical calculations and the experimental results on the pressure dependence of the direct and indirect transitions of *h*-BN indicate an enhancement of the indirect character of this material with increasing pressure. The energies of both direct and indirect transitions decrease with pressure, the indirect one exhibiting a faster decrease rate. This is in contrast with the pressure behavior of WSe_2_, which displays a positive pressure coefficient for the direct excitonic transition. Such positive pressure coefficient and the indirect transition being relatively insensitive to pressure make it possible to achieve a pressure-induced transition from direct to indirect emission in monolayer WSe_2_.^22^ However, for bilayer WSe_2_, the interlayer coupling strongly affects the pressure response of the Λ valley, which results in a rapid redshift of the indirect transition and leads to a further enhancement of the indirect character of bilayer WSe_2_ with increasing pressure.^22^ The pressure behavior of *h*-BN is different in that both direct and indirect transitions exhibit a negative pressure coefficient. Nevertheless, the effect of pressure also enhances its indirect character because the indirect transition energy decreases at a higher rate than the direct transition.

## Conclusions

We have investigated the pressure-induced shifts of direct and indirect excitonic transitions of the indirect-bandgap bulk *h*-BN layered crystal. Optical reflectance experiments have proven to be a sensitive tool for assessing the tuning of the excitonic transitions of *h*-BN by hydrostatic pressure. With increasing pressure, the indirect excitonic transition exhibits a redshift with a pressure coefficient of −36 ± 2 meV GPa^–1^, whereas the direct excitonic transition red-shifts at a somehow smaller rate (−26 ± 2 meV GPa^–1^). This different pressure behavior reinforces the indirect character of the bulk *h*-BN crystal with increasing pressure. A similar reinforcement was previously found in bilayer WSe_2_, while monolayer WSe_2_ exhibits a direct-to-indirect transition at moderate pressures (around 2.25 GPa). Given the negative pressure coefficient we find for the direct transition of *h*-BN, it seems unlikely that a direct-to-indirect transition could take place in direct-bandgap monolayer *h*-BN,^35^ although further investigations should be necessary to elucidate this point.

The reflectance experiments have revealed changes in the direct and indirect excitonic transitions that provide information about the electronic band shifts in the layered compound *h*-BN arising from the enhancement of interlayer interactions. Features in the reflectance spectra associated with direct and indirect excitonic transitions show a redshift with pressure. The analysis of these results on the basis of *ab initio* calculations indicates a significant lowering of the conduction band minimum at *M* and strong excitonic effects.

Excitonic effects tend to flatten the electron–hole dispersion, leading to different binding energies for the direct and indirect excitons. The direct exciton binding energy decreases significantly with pressure. The calculations show that the indirect excitonic transition energy decreases faster with pressure than the direct exciton transition energy, as corroborated by the reflectance measurements. Unlike other 2D materials such as WSe_2_, the pressure coefficient for the direct excitonic transition is also negative. Given the relevance of band engineering in strained layered materials, the data on *h*-BN band structure changes under compression presented here will be relevant for future research in novel applications of *h*-BN in layered heterostructures.
